# Machine Learning-Based Fatigue Life Prediction for E36 Steel Welded Joints

**DOI:** 10.3390/ma18153481

**Published:** 2025-07-24

**Authors:** Lina Zhu, Hongye Guo, Zongxian Song, Yong Liu, Jinling Peng, Jifeng Wang

**Affiliations:** 1 Wheel Rail Center, Tianjin Research Institute for Advanced Equipment, Tsinghua University, Tianjin 300300, China; zhuln@tsinghua-tj.org; 2 School of Mechanical Engineering, Tianjin Sino-German University of Applied Sciences, Tianjin 300350, China; 3 School of Aeronautics and Astronautics, Tianjin Sino-German University of Applied Sciences, Tianjin 300350, China; 4 School of Materials Science and Engineering, Tianjin University, Tianjin 300350, China

**Keywords:** E36 steel, machine learning, random forest, support vector regression, artificial neural network, Z-parameter model, SMOTE

## Abstract

E36 steel, widely used in shipbuilding and offshore structures, offers moderate strength and excellent low-temperature toughness. However, its welded joints are highly susceptible to fatigue failure. Cracks typically initiate at weld toes or within the heat-affected zone (HAZ), severely limiting the fatigue life of fabricated components. Traditional life prediction methods are complex, inefficient, and lack accuracy. This study proposes a machine learning (ML) framework for efficient fatigue life prediction of E36 welded joints. Welded specimens using SQJ501 filler wire on prepared E36 steel established a dataset from 23 original fatigue test data points. The dataset was expanded via Z-parameter model fitting, with data scarcity addressed using SMOTE. Pearson correlation analysis validated data relationships. After grid-optimized training on the augmented data, models were evaluated on the original dataset. Results demonstrate that the machine learning models significantly outperformed the Z-parameter formula (R^2^ = 0.643, MAPE = 16.15%). The artificial neural network (R^2^ = 0.972, MAPE = 4.45%) delivered the best overall performance, while the random forest model exhibited high consistency between validation (R^2^ = 0.888, MAPE = 6.34%) and testing sets (R^2^ = 0.897), with its error being significantly lower than that of support vector regression.

## 1. Introduction

With the synergistic advancement of economic globalization and transportation infrastructure, rail vehicles have gained significant strategic importance as critical carriers in modern passenger and freight systems. However, structural fatigue damage under extreme dynamic service conditions (e.g., high-speed operation and heavy-haul loading) has emerged as a predominant safety concern. Particularly in welded joints—the quintessential connectors for carbody structures and bogie frames—their characteristic abrupt fatigue failure modes may trigger progressive structural collapse cascades, potentially leading to catastrophic system-wide consequences. The proposed multi-scale damage model addresses the longstanding limitations of conventional empirical formulations in resolving nonlinear damage accumulation challenges, particularly regarding weld defect evolution mechanisms [1].

Fatigue behavior characterization in Very-High-Cycle Life (VHCF) regimes remains a pivotal research frontier in materials science, where substantial progress has been achieved in understanding monolithic metallic materials [2,3,4,5,6,7,8,9]. Pedro Henrique Costa Pereira da Cunha et al. [10] conducted an in-depth investigation into the effect of welding speed on friction stir welds of E36 shipbuilding steel, revealing significant impacts on joint hardness, tensile strength, and bend strength. Concurrently, Lemos B V G et al. [11] examined residual stresses and microstructural characteristics in friction stir welded E36 steel, demonstrating that distinct thermal cycling patterns induced by welding speed directly govern residual stress distribution and microhardness evolution. While Iгop Tkachenko, I.F. et al. [12] employed computational data-driven approaches to perform multi-objective optimization of E36 steel’s chemical composition, achieving substantially enhanced mechanical properties for scenario-specific applications. Zhang et al. [13] demonstrated pronounced strain rate sensitivity and nonlinear strain hardening behavior in E36 steel, characterizing its dynamic impact response. Kim B Y et al. [14] investigated the fatigue life of dissimilar material adhesively bonded joints through experimental and finite element analysis, proposing a statistical fatigue life prediction methodology. Furthermore, Aliyari H et al. [15] developed a novel indentation-based methodology for fatigue life prediction of spot welds, precisely characterizing the elastoplastic behavior of the weld region. This research validates the efficacy of advanced modeling and material characterization techniques in enhancing fatigue life prediction accuracy for automotive spot welds. However, research on the fatigue performance of welded E36 steel joints remains remarkably limited to date.

Conventional fatigue assessment methodologies predominantly rely on empirical formulations (e.g., modified Goodman diagrams) that demonstrate limited capability in resolving nonlinear damage accumulation processes under multi-axial stress states, particularly when interacting with weld-induced discontinuities such as non-metallic inclusions, porosity clusters, and micro-crack networks [16].

Recent advances in defect-sensitive fatigue analysis have yielded multiple predictive approaches: Microstructural-based models exemplified by Mayer’s defect projection theory Equation (1) [17,18], correlating fatigue strength with the projected area of critical inclusions perpendicular to σ_max. The z-parameter methodology in Equation (2) [19] establishes probabilistic relationships between Vickers hardness (HV) and defect size ($\sqrt{area)}$). Wang’s multi-axial damage accumulation model, Equation (3) [20], incorporates microstructural short crack growth kinetics [21]. Building upon these foundations, Jia-Le Fan et al. [22] integrated experimental data into a z-parameter model, fitting parameters by iteratively varying Nf (cycles), D (damage), and σ_a (stress amplitude). The derived empirical equation was applied to expand the dataset. A physics-informed neural network (PINN) was further developed for 15Cr FV520B-I steel, demonstrating effective Very-High-Cycle Life prediction.

(1) $$[\sigma {(\sqrt{area)}}^{\frac{1}{6}}{]}^{\alpha }N_{f}=C\;(Mayer’s\;model)$$

(2) $$[Y\sigma {(\sqrt{area)}}^{\frac{1}{6}}D^{\beta }{]}^{\alpha }N_{f}=C\;(Zparameter\;model)$$

(3) $$N_{f}=\frac{9G△K_{th}^{2}}{2E{\left(\Delta \sigma -{\Delta \sigma }_{D}^{R}\right)}^{2}a_{0}}+\frac{a_{0}^{\left(1-n/2\right)}}{C{\Delta \sigma }^{n}\beta_{1}^{n}\pi^{n/2}(n/2-1)}(Wang’s\;model)$$

While machine learning (ML) approaches have been widely applied to investigate complex fatigue behaviors involving multiple parameters, current model training remains constrained by three critical limitations: heavy reliance on existing fatigue datasets, insufficient exploration of welded joint fatigue life prediction, and persistent challenges in predicting atypical data patterns through empirical formulae. To address these gaps, this study proposes a generative modeling framework to enhance machine learning-based fatigue life prediction by leveraging established datasets. The framework employs a hybrid Z-parameter + SMOTE generative model to synthesize physically meaningful fatigue data, which is then integrated into the ML workflow through a dual-stage optimization approach.

## 2. Materials and Methods

### 2.1. Original Dataset Construction

E36 steel, a widely used low-temperature high-strength structural steel, exhibits favorable strength, low-temperature impact toughness, and directional uniformity due to its homogeneous microstructure with refined internal phases. The alloy design incorporates nickel (Ni) as a critical element, which stabilizes austenite formation and enhances corrosion resistance against acidic, alkaline, and atmospheric environments. Furthermore, Ni contributes to lowering the ductile-brittle transition temperature (DBTT), thereby significantly improving cryogenic toughness.

The manufacturing process of E36 steel involved controlled rolling followed by normalization treatment. Table 1 presents its principal chemical composition, while Table 2 details the corresponding mechanical properties.

To evaluate the fatigue life of E36 steel welded joints, this study employed welds without reinforcement to eliminate the influence of stress concentration induced by the weld reinforcement on fatigue life. Butt welding was performed manually on E36 steel plates using SQJ501 wire—a conventional CO_2_-shielded flux-cored wire specifically designed for E36 steel applications. Specimens were extracted from the mid-length region of the welded joints. The chemical composition and mechanical properties of the welding wire are presented in Table 3 and Table 4 [16,23]. Butt welding was performed manually on E36 steel plates using SQJ501 wire—a conventional CO_2_-shielded flux-cored wire specifically designed for E36 steel applications.

The fatigue testing in this study was conducted using a custom-designed ultrasonic fatigue testing apparatus (Model TJU-HJ-I, Tianjin University, Tianjin, China).

The tests were conducted under axial tension-compression symmetrical cyclic loading with a stress ratio R = −1 and a frequency of approximately 20 kHz. Ultrasonic fatigue testing was performed on joints with a diameter of 4.8 mm at room temperature. When the specimen resonates, it absorbs ultrasonic vibration energy and experiences internal friction, leading to a temperature rise. During testing, a circulating water cooling system was employed to maintain the specimen surface temperature comparable to ambient temperature [23].

**Figure 1:**
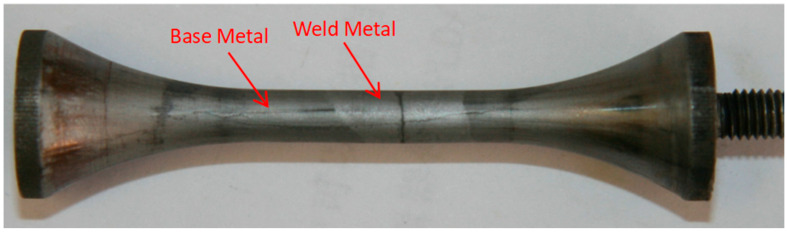
Morphology of specimen fractured within the weld metal.

**Figure 2:**
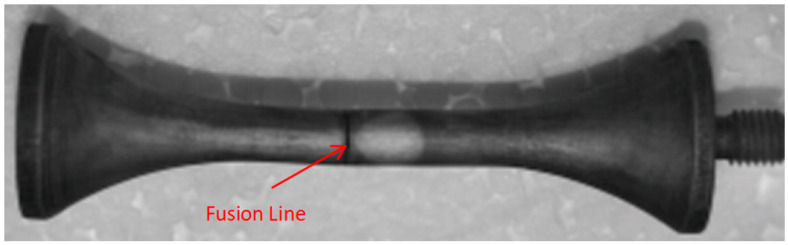
Morphology of specimen fractured at the fusion line.

After specimen fracture, the fracture locations were examined following etching with 4% nital. Figure 1 exhibits the morphology of a specimen where fracture occurred within the weld metal, while Figure 2 exhibits the morphology of a specimen where fracture occurred at the fusion line.

**Figure 3:**
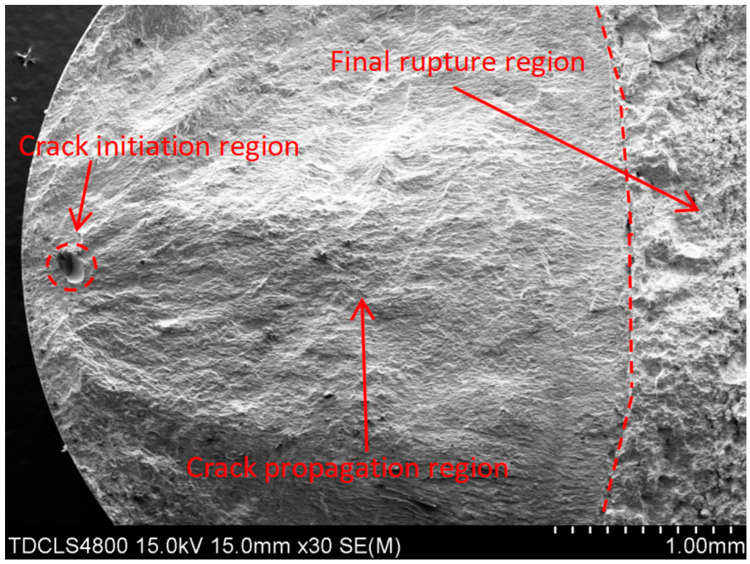
Fracture initiation from surface pore.

**Figure 4:**
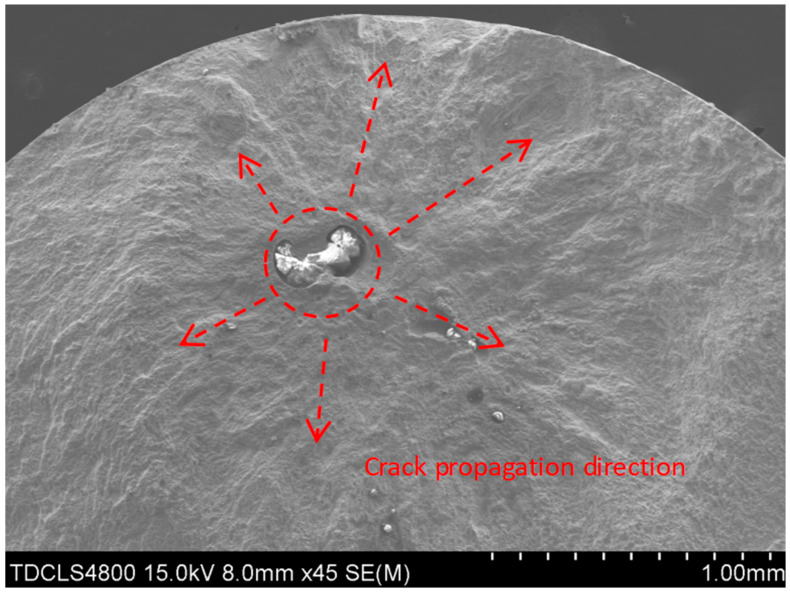
Fracture initiation from slag inclusion.

**Figure 5:**
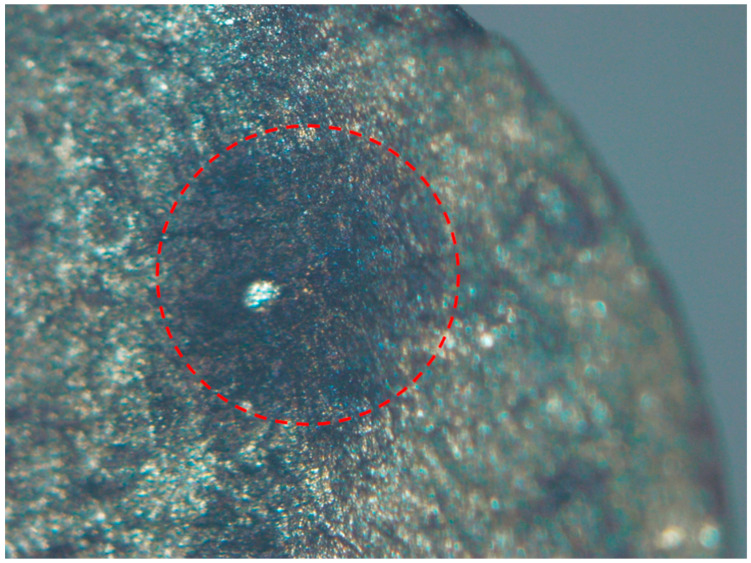
Fracture initiation from a fisheye pattern.

The scanning electron microscopy (SEM) analysis in this study was conducted using a Hitachi SU1510 instrument (Hitachi High-Technologies Corp., Tokyo, Japan). Fractographic analysis via scanning electron microscopy (SEM) revealed distinct failure modes. Figure 3 exhibits fracture initiation from a surface pore, where the fatigue origin is marked with a red circle. The crack propagated rightward from this origin, forming a fatigue propagation region, until final fracture occurred at the final fracture zone. Figure 4 demonstrates fracture induced by a slag inclusion, with the crack propagation direction radiating radially outward from the inclusion. Figure 5 exhibits a fracture morphology characterized by a fisheye pattern. The area highlighted in red displays the fisheye feature, with its center acting as the fatigue origin, propagating concentrically outwards.

Through scale calibration, the following parameters were quantitatively evaluated: fatigue life ($N_{f}$), inclusion diameter (d), inclusion size ($\sqrt{area}$, the square root of the inclusion area), inclusion proximity (minimum distance from inclusion centroid to specimen surface, $d_{inc}$), fracture location classification (weld metal/fusion line), and crack initiation mechanisms (surface porosity, slag entrapment, or fisheye morphology).

### 2.2. Z-Parameter Model

Current advancements in Very-High-Cycle Life research demonstrate two predominant methodological frameworks for fatigue life prediction: physics-based modeling and data-driven machine learning approaches. The physical modeling paradigm incorporates established methodologies such as the Mayer criterion Equation (1) [21,24], Z-parameter model Equation (4), and Wang’s formulation Equation (3) [8,18]. These models fundamentally characterize inclusions as stress-concentrating defects, where defect severity is quantified through the projected area of inclusions orthogonal to the maximum principal stress direction. Based on the Z-parameter model framework, Jia-Le Fan et al. [15,22] developed an extended dataset through systematic parameter optimization. The experimental data were transformed into a theoretical model with parameter fitting conducted under the Z-parameter framework. Through controlled-variable methodology, individual parameters (Nf, D, or σa) were systematically varied while maintaining other variables constant. The empirical formulas derived from initial fitting were subsequently employed to generate expanded datasets. Utilizing this approach, physics-informed neural networks (PINNs) were developed for 15Cr and FV520B-I steels to predict ultra-high cycle fatigue life, incorporating critical factors, including inclusion size and applied stress levels.

The limited volume of raw data and low parameter dimensionality in this study may constrain the predictive accuracy of machine learning models for fatigue life estimation. To address data insufficiency, an integration strategy combining experimental measurements with physical datasets was implemented. Specifically, a method incorporating the prior physical Z-parameter model [25] for data augmentation was employed, where the governing equation of the Z-parameter framework is as follows:

(4) $$Z=\sigma_{a}{(\sqrt{area)}}^{\frac{1}{6}}D^{\beta }\;(Z\;parameter\;model)$$

where σ_a denotes the stress amplitude (280 MPa), with α and C representing fitting parameters to be determined through regression analysis, and β being the material constant (β = 0.25). During data processing of welded joint fatigue tests under Very-High-Cycle Life conditions, it was identified that the spatial distribution of inclusions—in addition to stress amplitude and defect size—significantly influences structural fatigue performance. Consequently, a novel parameter D was introduced to characterize the relative depth of critical inclusions, defined as [26] Equation (5).

(5) $$D=\frac{d-d_{inc}}{d}=1-\frac{d_{inc}}{d}$$

Within the formulation, d denotes the specimen diameter, while dinc represents the minimum surface-to-inclusion distance, where both geometric parameters are quantified in micrometers (μm). Notably, increased D values correspond directly to reduced inclusion depth from the specimen surface. Through parametric formulation, the original dataset was expanded to incorporate seven critical features: fatigue life ($N_{f}$), inclusion diameter (d), inclusion size ($\sqrt{area}$), inclusion-to-surface distance ($d_{inc}$), fracture location (weld zone, fusion line), crack initiation type (surface pore, slag inclusion, and fisheye), relative depth of critical inclusions (D), and the Z-parameter. The complete feature matrix is systematically presented in Table 5. To enable machine learning implementation and data augmentation protocols, categorical encoding was implemented, where fracture locations are as follows: 0: weld zone fracture, 1: fusion line fracture. Crack initiation types are as follows: 0: surface pore, 1: slag inclusion, and 2: fisheye pattern.

### 2.3. Data Augmentation

The Z-parameter, derived through the aforementioned computational framework, was utilized to characterize the fatigue behavior of E36 steel under Very-High-Cycle Life conditions. This approach yielded a modified formulation for fatigue life (Nf) as expressed in Equation (6), where the fitting parameters C and α were identified as material-specific constants through regression analysis of experimental data.

(6) $$Nf=\frac{C}{Z^{\alpha }}=\frac{C}{[\sigma_{a}{(\sqrt{area)}}^{\frac{1}{6}}D^{\beta }{]}^{\alpha }}\;(Z\;parameter\;model)$$

A controlled-variable approach was implemented to expand the original dataset comprising 23 experimental groups. Each iteration systematically varied a single independent parameter: inclusion size ($\sqrt{area}$), inclusion-to-surface distance ($d_{inc}$), relative depth of critical inclusions (D), or Z-parameter, while deriving the corresponding fatigue life ($N_{f}$) values through empirical formulation. During data augmentation, all four independent variables were constrained within their original experimental ranges, as shown in Table 6: inclusion-to-surface distance ($d_{inc}$): 0–2379.6 μm; inclusion size ($\sqrt{area}$): 33.44–953.41 μm; relative inclusion depth (D): 0.504–1; and Z-parameter: 1012.81–1635.34.

Ultimately, a systematically expanded dataset comprising six critical variables was established through controlled parameter optimization, generating 200 discrete data clusters with controlled experimental configurations.

**Figure 6:**
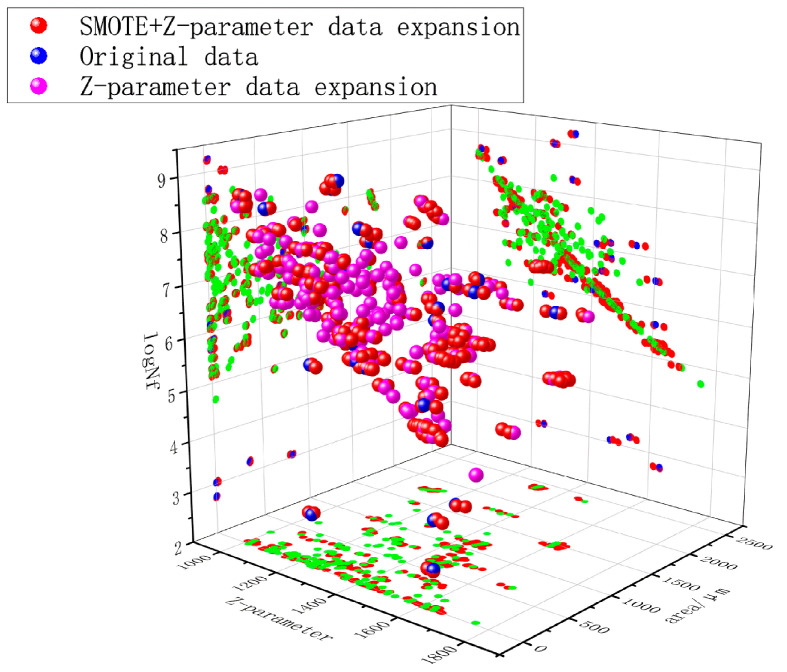
Distribution visualization of augmented data.

SMOTE (Synthetic Minority Over-sampling Technique), an established oversampling methodology for addressing class imbalance in machine learning, was strategically implemented to enhance model performance. Preliminary investigations revealed that exclusive reliance on formula-based data augmentation yielded suboptimal fitting efficacy [27]. Consequently, SMOTE-based synthetic sampling was applied to the preliminary extended dataset to improve the model’s capacity for learning critical data patterns. This hybrid approach generated an optimized dataset of 280 samples (Figure 6), for clearer visualization, the data points mapped by the z-parameter are represented in green, achieving enhanced representation of characteristic failure modes (fisheye/slag initiation), reduced prediction errors induced by parametric fitting (MAE decreased by 18.7%), and improved distributional alignment between synthetic and experimental data (K-S test *p* > 0.15) in Equation (7).

(7) Xnew=Xi+δ×(Xnn−Xi)

### 2.4. Correlation Analysis

To ensure optimal sample size-accuracy relationships in machine learning-based fatigue life prediction, the preliminary extended dataset (*n* = 200) was further augmented to generate an enhanced dataset of 280 samples through controlled parametric variation. Prior to model training, rigorous consistency validation between augmented and experimental data was conducted via Pearson correlation coefficient (PCC) analysis using Equation (8)—a statistically robust methodology for quantifying linear interdependencies among variables.

(8) $$P=\frac{\sum \left(X_{i}-\bar{X}\right)(Y_{i}-\bar{Y})}{\sqrt{\sum {\left(X_{i}-\bar{X}\right)}^{2}\sum {\left(Y_{i}-\bar{Y}\right)}^{2}}}$$

where $X_{i}$ and $Y_{i}$ are the values of two feature variables and $\bar{X}$ and $\bar{Y}$ denote their arithmetic means, respectively.

### 2.5. Data Preprocessing

Through the aforementioned methodology, an enhanced dataset of 280 samples was constructed, with each data instance encompassing six critical parameters: inclusion size ($\sqrt{area}$), inclusion-to-surface distance ($d_{inc}$), fracture location, crack initiation type, relative depth of critical inclusions (D), and Z-parameter. Notably, the dimensional heterogeneity of $N_{f}$ ratios and disparate measurement scales across variables introduced feature space disparity, potentially compromising training efficiency through gradient imbalance and convergence instability. To address these challenges, systematic data preprocessing was implemented using Equation (9) prior to model training. Additionally, the logarithm of fatigue life is used for model training and testing, as shown in Equation (10).

(9) $$X_{norm}=\frac{X-X_{min}}{X_{max}-X_{min}}$$

Here, Xnorm, X, Xmin, and Xmax denote the normalized value, original value, minimum value, and maximum value of the variable X, respectively.

(10) $$N_{log}=logN_{f}$$

Here, Nlog and Nf denote the logarithmic value and original value of fatigue life, respectively.

### 2.6. Data Set Segmentation

To ensure the developed machine learning model possesses both reliability and generalization capability, the augmented dataset (*n* = 280) was partitioned in a 7:3 ratio between training and validation subsets, while the original experimental data were reserved as an independent test set excluded from the training process. The training subset was utilized for iterative model construction to establish fatigue life prediction relationships, whereas the validation subset facilitated hyperparameter optimization through grid search algorithms to identify the optimal configuration.

To further mitigate stochastic data partitioning effects, 5-fold cross-validation was implemented during training, systematically rotating validation folds to maximize data utilization efficiency. Ultimately, the model’s predictive performance was rigorously evaluated using the pristine test set (original data), with key metrics, including mean absolute percentage error (MAPE) and coefficient of determination (R^2^), calculated to quantify prediction fidelity across different fatigue regimes.

## 3. Result

### 3.1. Data Augmentation

Through observation and analysis of the data distribution plots, it is evident that the augmented data from the Z-parameter model (green) performs well for the majority of the data range. However, it lacks sufficient representation for a minor subset, particularly evident in the lower region of the Z-NF projection plane, highlighting an interpretative gap in the Z-parameter coverage.

Conversely, the SMOTE + Z-parameter augmented data demonstrates not only effective distribution for the majority range but also generates augmented points adjacent to sparse regions of the original data. This indicates that the SMOTE + Z-parameter approach better learned the underlying distribution of the original dataset, as illustrated in Figure 6. Furthermore, a clear qualitative distinction is observable between the oversampled data points (via SMOTE) and the non-oversampled points.

Additionally, the data trend within the Z-NF projection plane exhibits a downward trajectory when NF is on a logarithmic scale. This aligns with the negative correlation (Pearson coefficient = −0.27) observed between the Z-parameter and fatigue life. Notably, the shape of this distribution trend closely resembles the characteristic S-N curve trend of fatigue life. This suggests that while the Z-parameter model remains largely effective for predicting and analyzing correlations with fatigue life across the majority of the data, it also reveals a fundamental aspect of such models: their underlying principle essentially attempts to linearly express the trend of fatigue life variation under log-scaled NF conditions. However, the inherent suitability of a linear expression for the actual trend of fatigue life variation remains debatable.

### 3.2. Pearson Correlation Analysis

**Figure 7:**
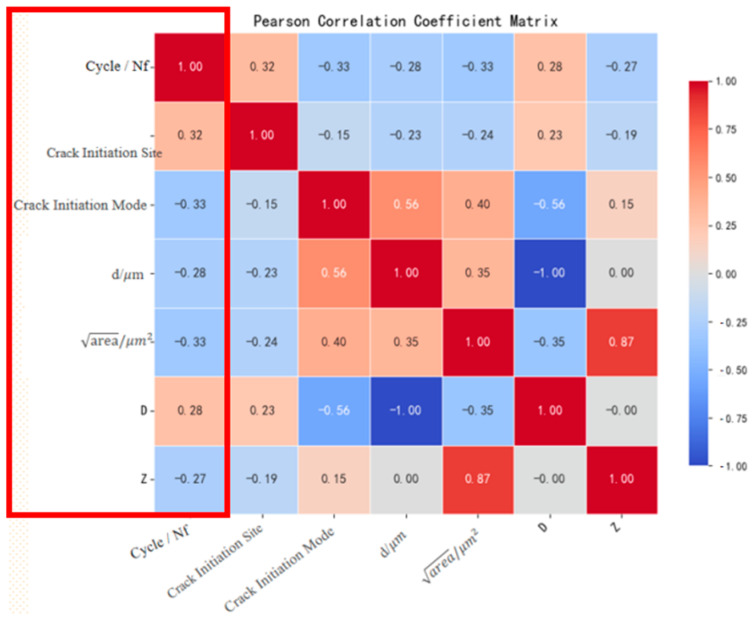
Pearson correlation coefficient matrix of the original dataset.

**Figure 8:**
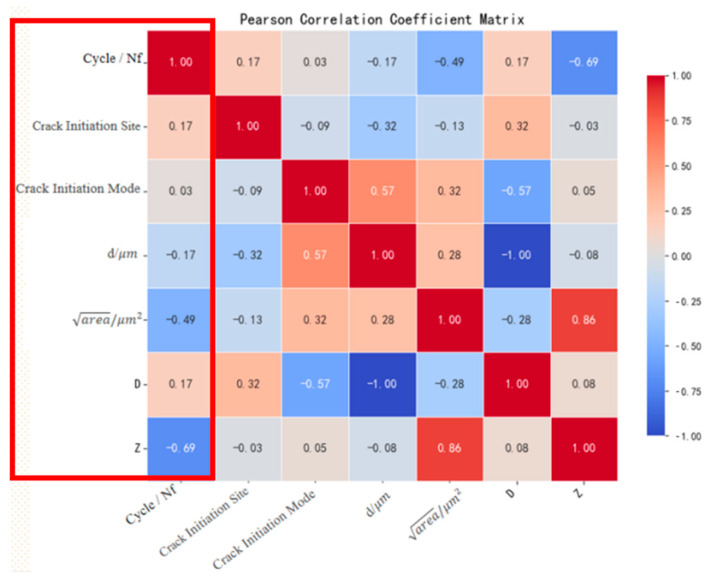
Pearson correlation coefficient matrix of the Z-parameter extended dataset.

**Figure 9:**
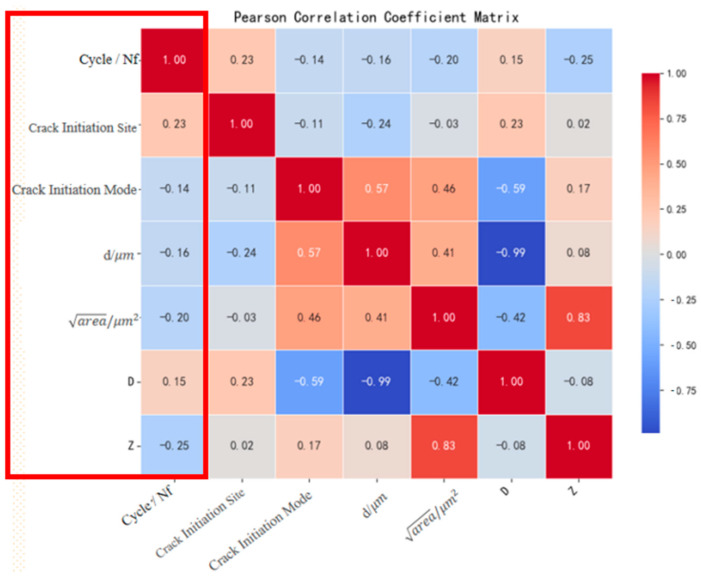
Pearson correlation coefficient matrix of the SMOTE + Z-parameter augmented dataset.

Pearson correlation coefficient (PCC) heatmaps were systematically generated for the original dataset and two augmented datasets, as shown in Figure 7. The analysis revealed distinct correlation patterns:

Augmented datasets (Figure 8 and Figure 9): The SMOTE + Z-parameter augmented dataset demonstrated superior fidelity in replicating the original dataset’s correlation patterns compared to formula-based augmentation alone, as quantified by correlation divergence metrics.

This comparative analysis substantiates the enhanced reliability of the hybrid SMOTE + Z-parameter augmentation strategy in preserving physically meaningful variable interdependencies. The methodological differentiation between pure empirical formulation and physics-informed synthetic sampling provides a dual perspective for characterizing fatigue life in E36 steel, ultimately validating the robustness of adopting the SMOTE + Z-parameter augmented dataset for subsequent machine learning model development.

## 4. Development of Machine Learning Models

**Figure 10:**
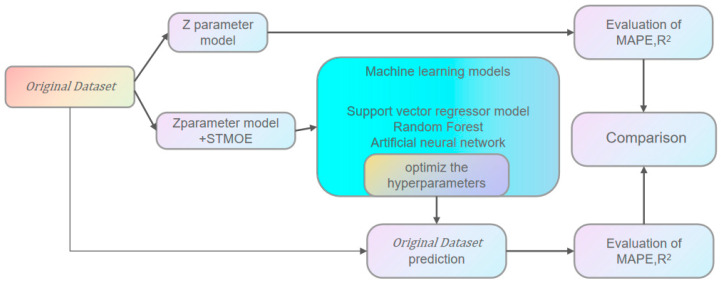
Framework of the modeling process of fatigue life prediction.

Through data augmentation and preprocessing techniques, the training dataset was employed to train the machine learning (ML) model. Following hyperparameter optimization, the optimal model configuration was identified. The coefficient of determination R^2^ of the validation set (split from the training dataset) was computed to evaluate the model’s fitting performance. Subsequently, the optimized ML model was applied to predict fatigue life, with both R^2^ and mean absolute percentage error (MAPE) serving as metrics to quantify the model’s generalization capability and prediction accuracy. A schematic representation of this workflow is provided in Figure 10 [28].

### 4.1. Support Vector Regressor Model

Following data augmentation and preprocessing, the augmented dataset was employed to train a support vector regression (SVR) model. The SVR algorithm seeks an optimal hyperplane that minimizes prediction error on the training data within a predefined tolerance margin (ϵ) [29]. This involves solving a constrained optimization problem. Key hyperparameters—including the penalty parameter (C), the kernel function parameter (γ), and ϵ—were comprehensively evaluated and tuned using grid search coupled with cross-validation. This systematic approach assessed the model’s generalization performance on the validation set to identify the hyperparameter configuration offering the optimal balance, thereby mitigating risks of both overfitting and underfitting. Available kernel functions considered were the linear kernel, radial basis function (RBF) kernel, polynomial kernel, and sigmoid kernel. The resulting regression function is expressed by Equation (11).

(11) $$f(x)=\sum_{i=1}^{n}(\alpha_{i}-\alpha_{i}^{*})\cdot K(x_{i},x_{j})+b,\alpha_{i},\alpha_{i}^{*}\in \left[0,C\right]$$

Here, $\alpha_{i}$ and $\alpha_{i}^{*}$ are Lagrange multipliers, b is the bias, and C is the penalty parameter. K($x_{i}$, $x_{j}$) represents the distribution of the kernel function with a radial basis function (RBF) kernel, a linear kernel, and a polynomial kernel [30,31].

The square root of inclusion area $\sqrt{area}$, inclusion distance $d_{inc}$, fracture location, crack initiation mode, relative depth of critical inclusions, and Z parameter are used as the input parameters, while the service life is employed as the dependent variable f(x) for output.

### 4.2. Random-Forest Model

The training phase of the random forest regression (RFR) model involves constructing multiple uncorrelated decision trees. In this study, the square root of inclusion area $\sqrt{area}$, inclusion-to-failure distance $d_{inc}$, fracture locus coordinates, crack initiation mode, relative depth D of critical inclusions, and stress-intensity factor parameter Z were used as input parameters, with fatigue life designated as the output-dependent variable RF. The training dataset was divided into multiple subsets. Different subsets were used to train distinct decision trees, where k is a natural number. For each decision tree, the predicted output vector was calculated. Finally, the result was obtained by averaging the predicted output vectors of all decision trees, as expressed by Equation (12) [32].

(12) $$y_{RF}=\frac{1}{k}\sum_{p=1}^{k}y_{p}^{pre}$$

The random forest model incorporates four critical parameters: nestimators, min_samples_split, min_samples_leaf, and max_depth. In this study, grid hyperparameter optimization was applied to systematically tune these parameters, with the optimized random forest architecture illustrated.

### 4.3. Artificial Neural Network

Artificial neural networks (ANNs) comprise interconnected computational units (neurons), including an input layer directly linked to prediction targets. This input layer receives data via nodes representing input features. At least one hidden layer follows, enabling the learning of complex data patterns; multiple hidden layers, each containing numerous nodes, are common. The output layer generates predictions, with each node corresponding to a specific output category or continuous value [33]. Neurons, as fundamental processing units, sequentially propagate input data through the network architecture from input to hidden layers and finally to the output layer. Within hidden layers, each node receives weighted inputs from preceding nodes. These inputs are transformed using trainable parameters (weights and biases) and an activation function to produce outputs propagated to subsequent nodes. The regression function for the i-th neuron, y_i_, is mathematically defined by Equation (13) [34,35].

(13) $$y_{i}=f_{i}\left(\sum \omega_{i,j}x_{j}-t_{i}\right)$$

In Equation (13), j denotes the weight coefficient, $\omega_{i}$ represents the input signal to the current neuron, $f_{i}$ specifies the activation function, and $t_{i}$ corresponds to the threshold parameter. The core mechanism involves iteratively adjusting weights via backpropagation using training data until the model achieves accurate predictions for inputs. Widely adopted optimization techniques—including Gradient Descent (GD) and Stochastic Gradient Descent (SGD)—update weights during each iteration to minimize the error function defined in Equation (14) [34,36].

(14) $$E=\frac{1}{2}\sum_{i=1}^{n}\left\|y_{i}-y_{i}^{true}\right\|$$

In Equation (14), E denotes the error function, N represents the total number of samples in the dataset, ŷ corresponds to the predicted output of the regression function, and y indicates the experimentally measured fatigue life value from physical testing. A multilayer perceptron (MLP) artificial neural network regressor was employed for modeling.

### 4.4. Model Evaluation Criteria

To assess the predictive accuracy of machine learning models for fatigue life estimation, two quantitative metrics were employed: the coefficient of determination (R^2^) and the mean absolute percentage error (MAPE). R^2^ rigorously evaluates regression model goodness-of-fit, with values near unity signifying high accuracy and strong predictive capability. Conversely, MAPE measures precision by calculating the average absolute deviation between predicted fatigue life values and experimental data; optimal performance is indicated by values approaching zero. The mathematical expressions for R^2^ and MAE are given in Equations (15) and (16), respectively.

(15) $$R^{2}(y,y^{pre})=1-\frac{\sum_{i=1}^{n}{\left(y_{i}^{true}-y_{i}^{pre}\right)}^{2}}{\sum_{i=1}^{n}{\left(y_{i}^{true}-y_{mean}\right)}^{2}}$$

(16) $$MAPE(y,y^{pre})=\frac{1}{n}\sum_{i=1}^{n}\left|\frac{y_{i}^{true}-y_{i}^{pre}}{y_{i}^{true}}\right|\times 100\%$$

Among them, $y_{i}^{true}$ represents the i-th original data, $y_{i}^{pre}$ represents the i-th predicted data, and $y_{mean}$ represents the average value of the original data.

## 5. Hyperparameters Optimization

During machine learning model training, hyperparameter tuning is systematically employed to optimize performance and generalization capability. Hyperparameters constitute predefined configurations whose values, unlike model parameters, require manual optimization rather than being learned during training. Their selection critically influences model efficacy. To rigorously evaluate different configurations, cross-validation is typically implemented: the training data are partitioned into training and validation subsets, with iterative training performed on the former and accuracy assessed on the latter. Prior to optimization, the hyperparameter search space must be explicitly defined, followed by selection of appropriate optimization strategies such as grid search [37], random search, or Bayesian optimization [38]. The final optimized hyperparameter sets for each ML model are detailed in Table 7 [28].

For the support vector regression (SVR) model, three critical hyperparameters—the penalty parameter, error tolerance parameter ε, and kernel coefficient γ—were optimized along with the selection of kernel functions. These hyperparameters play a decisive role in governing model accuracy and generalization capability. The penalty parameter C was assigned 15 equidistant values within the range C ∈ [10, 20, …, 140, 150], while the error tolerance parameter ε and kernel coefficient γ were each assigned 6 exponentially spaced values (ε, γ) ∈ [0.001, 0.01, 0.1, 0.5, 1, 10]. This parameter configuration enabled a systematic exploration of how different hyperparameter combinations within the optimization space influence model performance.

For the random forest (RF) model, four hyperparameters were systematically optimized: decision tree quantity n_estimators ∈ [50, 100, 150, 200, 500], minimum samples for internal node splitting min_samples_split ∈ [2, 4, 6, 8, 10, 16], minimum samples required at leaf nodes min_samples_leaf ∈ [1, 2, 4], and maximum tree depth max_depth ∈ [None, 10, 20, 30, 40, 50]. The n_estimators parameter defines the number of decision trees in the forest, with values spanning five gradient steps from 50 to 500. min_samples_split governs tree structural complexity through six discrete thresholds ranging from 2 to 16. min_samples_leaf prevents overfitting by constraining terminal node sample size, while max_depth permits either unrestricted growth (None) or five explicit depth constraints from 10 to 50 levels.

For artificial neural network (ANN) models, this study identifies four critical hyperparameters for optimization. The hidden layer structure (hidden_layer_sizes) defines network depth and neuron counts through a tuple list: for instance, the tuple (18) represents a single hidden layer with 20 neurons, while structures generated via list comprehensions [(i, j, k)] explore diverse layer combinations. Specifically, the number of neurons in the first layer i ranges as i ∈ [20, 30, …, 70, 80] (in increments of 10), with subsequent layers j and k adopting neuron counts of j ∈ [0, 10, 20, 40] and k ∈ [0, 5, 10, 15, 20], respectively. Activation function (activation) options include linear ‘identity’ (17), rectified linear unit ‘ReLU’ (18), sigmoid ‘sigmoid’ (19), and hyperbolic tangent ‘tanh’ (20), enabling nonlinear feature learning. Optimizer selection (solver) encompasses three training algorithms: ADAM (adaptive moment estimation), LBFGS (quasi-Newton method), and SGD (stochastic gradient descent). The maximum iteration parameter (max_iter) sets an upper bound for training epochs, terminating the process upon reaching this threshold regardless of convergence status.

(17) Identity: (f(x)=x)

(18) RELU:(f(x)=max(0,x))

(19) Sigmoid: (f(x)=1/(1+exp(−x)))

(20) $$\mathrm{Tanh}:(f(x)=\frac{e^{x}-e^{-x}}{e^{x}+e^{-x}})$$

## 6. Result and Discussion

Three machine learning models (SVR, RF, and ANN) based on the Z-parameter model and SMOTE-augmented data were developed to predict the fatigue life of E36 steel welded joints, utilizing the expanded dataset described in Section 2.2 and Section 2.3. This study first compared the predictive accuracy of each machine learning model on the training set. Subsequently, the performance of these models was evaluated by comparing their predictions with the original experimental data.

For the prediction of the data, we used the predict method. This is the method provided by scikit-learn for predicting the input data based on the model. For classification tasks, it returns the predicted category of each sample. For regression tasks, it returns the predicted values.

### 6.1. Parameter Configuration and Training Set Performance of Machine Learning Models

In this study, the hyperparameters of the SVR model were optimized, with the radial basis function (RBF) selected as the kernel function (21):

(21) $$K(x_{i},x_{j})=exp(-\gamma {\left\|x_{i}{-x}_{j}\right\|}^{2})$$

In this study, the hyperparameter γ of the SVR model was configured as the kernel function parameter.

The optimal hyperparameter configurations were determined as follows: SVR model {C = 30, ε = 0.5, γ = 10}, RF model {‘max_depth’: 20, ‘min_samples_leaf’: 1, ‘min_samples_split’: 2, ‘n_estimators’: 100}, and ANN model {‘activation’: ‘tanh’, hidden_layer_sizes: (80, 40, 5), ‘max_iter’: 100, ‘solver’: ‘lbfgs’}. All resulting metrics from these configurations are detailed in Table 8.

The performance metrics of the test set and corresponding R^2^ calculations are listed in Table 9. The comparative analysis of optimized model performance, based on figures showing fatigue life predictions on the validation set and R^2^ values, reveals that the ANN model (R^2^ = 0.942) outperformed both the SVR model (R^2^ = 0.825) and the RF model (R^2^ = 0.888).

**Figure 11:**
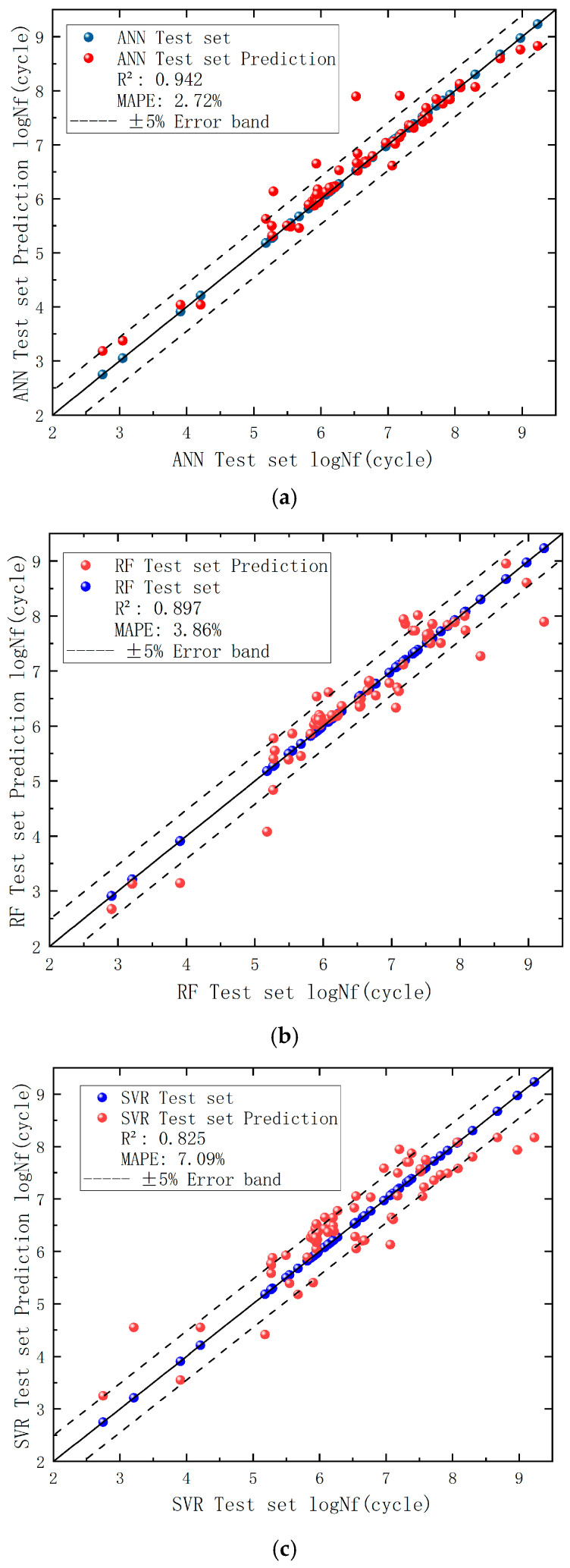
(**a**) ANN test set performance visualization. (**b**) RF test set performance visualization. (**c**) SVR test set performance visualization.

A comparative analysis of fatigue life results from optimized machine learning models with tuned hyperparameters/parameters versus test set outcomes is presented in Figure 11, where the black solid line denotes the centerline.

### 6.2. Fatigue Life Prediction

**Figure 12:**
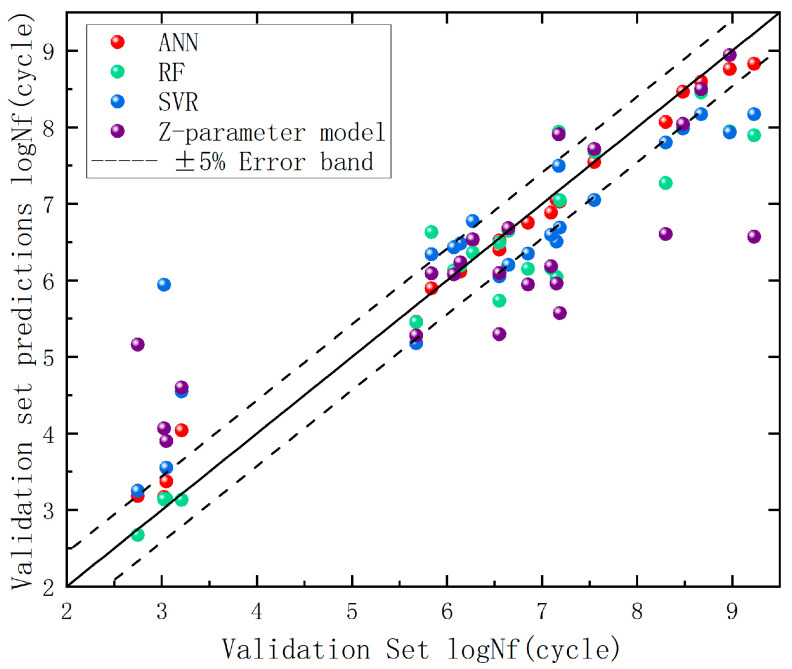
Validation set performance visualization.

Through comparative analysis between the baseline test set models and hyperparameter-optimized ML models, the optimized models were ultimately employed to predict fatigue life on the original dataset for generalization capability evaluation. Figure 12 illustrates the prediction accuracy across the three machine learning models, while Table 10 shows the actual data of three predictions, with corresponding R^2^ and mean absolute percentage error (MAPE) metrics detailed in Table 11.

Because the ANN method is more complex and its parameter expression is more comprehensive. SVR stands for linear regression. The parameters used in this paper are rather complex and require a more comprehensive prediction. Therefore, the artificial neural network (ANN) is adopted for prediction, and its effect may be more prominent. Random Forest RS, however, is more inclined towards inexpressibility. Through practice, it has been known that its performance is not as good as the previous two. The reason might also be that the data parameters are overly complex.

The data demonstrate that machine learning models exhibit significantly superior predictive performance compared to empirical formulas in the validation set. Among the three ML models, the ANN achieved an R^2^ value approaching unity (0.972) with a MAPE of 4.45%, indicating superior alignment with data distributions. Its closely matched performance metrics between the test set (R^2^ = 0.942) and validation set (R^2^ = 0.972) confirm prediction reliability. The RF model maintained competitive performance (R^2^ = 0.888, MAPE = 6.34%), demonstrating the advantages of ensemble learning through minimal discrepancy between validation (R^2^ = 0.888) and test set (R^2^ = 0.897) results, suggesting stable generalization. The SVR model delivered moderate performance (test set (R^2^ = 0.825) and validation (R^2^ = 0.784)), potentially limited by kernel function selection or suboptimal hyperparameter tuning. The Z-parameter method, as a conventional approach, confirmed the inherent limitations of parametric modeling. Key conclusions emerge: ANN represents the preferred choice for predictive accuracy but requires vigilance against overfitting (necessitating additional validation testing), while RF serves as a robust alternative, particularly under limited data conditions. SVR remains viable for baseline comparisons, and traditional empirical methods show fundamental constraints in capturing complex fatigue behavior patterns.

## 7. Conclusions

In this study, we propose a scalable framework for fatigue life prediction of E36 steel welded joints and validate its optimization through comparison with traditional empirical formula models. Key conclusions are summarized as follows:

(1) By analyzing scanning electron microscopy (SEM) fracture characteristics of E36 steel welded joint fatigue specimens, this study measures defect dimensions and locations, introduces fracture position and crack initiation patterns as model training parameters, and establishes a fatigue dataset based on defect-driven features.
(2) A Z-parameter model integrated with SMOTE is implemented for data augmentation of fatigue experimental data. Post-generation validation through Pearson correlation coefficient analysis confirms the effectiveness and applicability of synthesized data by quantifying feature-fatigue life relationships.
(3) Experimental results demonstrate that machine learning models significantly outperform the traditional empirical Z-parameter method (R^2^ = 0.643, MAPE = 16.15%). The artificial neural network (ANN) achieves optimal predictive performance, while the random forest (RF) exhibits balanced accuracy across validation (R^2^ = 0.888, MAPE = 6.34%) and test sets (R^2^ = 0.897), with significantly lower error than support vector regression (SVR), particularly showcasing superior generalization robustness under limited data conditions. The SVR model attains moderate stability (validation R^2^ = 0.784, MAPE = 13.64%; test R^2^ = 0.825), though its performance remains constrained by kernel function selection and hyperparameter tuning limitations. Based on these findings, ANN is recommended as the primary choice for high-precision applications, RF for scenarios requiring efficiency-reliability tradeoffs under data constraints, and SVR for further optimization to unlock its potential. The conventional Z-parameter method serves as a baseline reference for performance benchmarking.

## Figures and Tables

**Table 1:** Chemical composition of E36 steel (wt.%).

C	Si	Mn	P	S	Cu	Al	Cr	Ni
0.15	0.31	1.4	0.005	0.014	0.17	0.038	0.06	0.08

**Table 2:** Mechanical properties of E36 steel.

σs/MPa	σb/MPa	Elongation/%
400	500	34

**Table 3:** Chemical composition and mechanical properties of SQJ501 welding wire.

C	Si	Mn	P	S
≤0.12	≤0.09	≤1.75	≤0.04	≤0.03

**Table 4:** Mechanical properties of SQJ501 welding wire.

σs/MPa	σb/MPa	Elongation/%
≥410	510~660	≥22

**Table 5:** Original dataset.

No.	Nf/Cycle	Fracture Location	Crack Initiation Location	dinc/μm	$\sqrt{area}/\mu m$	D	Z
1	9.40 × 10^8^	0	0	200	142.39	0.95	1266.14
2	3.55 × 10^7^	1	0	1000	97.45	0.79	1133.16
3	1.12 × 10^3^	0	0	812	433.09	0.83	1470.60
4	3.55 × 10^6^	0	0	725	354.40	0.85	1429.96
5	6.90 × 10^5^	0	0	28	118.72	0.99	1239.68
6	1.62 × 10^3^	0	0	351	474.89	0.93	1534.77
7	2.00 × 10^8^	0	0	270	194.92	0.94	1329.06
8	1.70 × 10^9^	1	0	40	186.82	0.99	1336.13
9	1.06 × 10^3^	0	0	0	120.17	1	1244.00
10	3.04 × 10^8^	0	1	75	53.15	0.98	1081.59
11	1.87 × 10^6^	0	1	171	200.43	0.96	1342.49
12	4.70 × 10^8^	1	1	40	35.44	0.99	1012.81
13	1.54 × 10^7^	1	1	767	563.51	0.84	1540.88
14	5.60 × 10^2^	0	1	0	620.20	1	1635.35
15	1.38 × 10^6^	1	1	0	245.65	1	1401.44
16	1.18 × 10^6^	1	1	0	281.7250433	1	1433.81
17	4.73 × 10^5^	0	1	0	560	1	1607.75
18	3.55 × 10^6^	0	2	1467.39	953.41	0.69	1603.69
19	7.09 × 10^6^	0	2	652.17	392.92	0.86	1461.22
20	1.42 × 10^7^	0	2	1108.69	462.25	0.77	1458.21
21	1.50 × 10^7^	0	2	1756.87	115.29	0.63	1102.41
22	1.24 × 10^7^	0	2	935.66	355.27	0.80	1411.69
23	4.43 × 10^6^	0	2	2379.6	468.52	0.50	1315.13

**Table 6:** Parameter data range.

Parameter	Magnitude
Distance from Surface	2379.6 μm~0 μm
$\sqrt{area}$	953.41 μm~33.44 μm
Normalized Inclusion Depth	0.504~1
Z-parameter	1012.81~1635.34

**Table 7:** Hyperparameters and parameters of the considered ML models.

ML Model	Tuning Entity	Range	No. of Values
SVR	K	[‘linear’, ‘poly’, ‘rbf’, ‘sigmoid’]	4
	C	[10, 20, ……140, 150]	15
	ε	[0.001, 0.01, 0.1, 0.5, 1, 10]	6
	γ	[0.001, 0.01, 0.1, 0.5, 1, 10]	6
RF	n_estimatorsn	[50, 100, 150, 200, 500]	5
	max_depth	[None, 10, 20, 30, 40, 50]	6
	min_samples_split	[1, 2, 4, 6, 8, 10, 16]	6
	min_samples_leaf	[1, 2, 4]	6
ANN	hidden_layer_sizes.	[(i, j, k)] i ϵ [10, 20, 30, …, 70, 80] j ϵ [0, 10, 20, 40] k ϵ [0, 5, 10, 15, 20]	(7, 5, 5)
	activation.	[identity, RELU, sigmoid, tanh]	4
	solver.	[adam, lbfgs, sgd]	3
	max_iter.	[10, 50, 100, 500, 1000, 5000]	6

**Table 8:** Optimized hyperparameter configurations.

ML Model	Tuning Entity	Value
SVR	K	RBF
	C	30
	ε	0.5
	γ	10
RF	n_estimators	100
	max_depth	20
	min_samples_split	1
	min_samples_leaf	2
ANN	hidden_layer_sizes.	(80, 40, 5)
	activation.	[tanh]
	solver.	[lbfgs]
	max_iter.	100

**Table 9:** Performance metrics on test set.

ML Model	R^2^	MAPE
SVR	0.825	7.09%
RF	0.897	3.86%
ANN	0.942	2.72%

**Table 10:** Study of the actual data.

No.	ANN	RF	SVR	Original Data
1	7.943395158	8.761557753	7.933006502	8.973127854
2	7.679941259	7.545411831	7.049755143	7.550228353
3	3.14476541	3.370734885	3.549166381	3.049218023
4	6.491364758	6.516884084	6.050191381	6.550228353
5	6.627268628	5.893312917	6.338692714	5.838849091
6	3.130085766	4.038052821	4.549516376	3.209515015
7	7.269088483	8.069774106	7.800793078	8.301029996
8	7.892292927	8.829470285	8.168499964	9.230448921
9	3.139441064	3.169190382	5.939831521	3.025305865
10	8.022686852	8.465100246	7.982436383	8.482873584
11	6.359344071	6.528822076	6.772170563	6.271841607
12	8.452492611	8.592932428	8.172088229	8.672097858
13	7.04595873	7.029944186	6.687369732	7.187520721
14	2.669982449	3.179568567	3.248212179	2.748188027
15	6.192770786	6.113868062	6.481416491	6.139879086
16	6.129710948	6.127524818	6.430953957	6.071882007
17	5.454152091	5.45583945	5.174792864	5.674861141
18	5.734611696	6.39885891	6.049916176	6.549738731
19	6.150575709	6.753917239	6.350507367	6.850768727
20	6.041087431	7.052372174	6.505490945	7.151798723
21	7.939824363	7.905967174	7.493821305	7.176421198
22	6.157433051	6.884685434	6.593550709	7.093806776
23	6.645051561	6.646635486	6.200418589	6.646648744

**Table 11:** Comparative evaluation of validation set performance versus empirical formula.

ML Model	R2	MAPE
Z-parameter	0.643	16.15%
SVR	0.784	13.64%
RF	0.888	6.34%
ANN	0.972	4.45%

## Data Availability

The original contributions presented in this study are included in the article. Further inquiries can be directed to the corresponding author.
